# T follicular helper cells are elevated in a rat model of autoimmune myocarditis

**DOI:** 10.1002/2211-5463.12894

**Published:** 2020-06-11

**Authors:** Qi Xue, Yuan Ma, Lihong Wang, Hong Shao

**Affiliations:** ^1^ Department of Cardiology People’s Hospital of Hangzhou Medical College Zhejiang Provincial People’s Hospital Hangzhou China

**Keywords:** B cells, CXCL13, experimental autoimmune myocarditis, interleukin‐21, T follicular helper cells

## Abstract

Myocarditis is an inflammatory disease of the myocardium that is associated with immune dysfunction. Earlier studies have suggested that T helper 1/2 cell imbalance plays an important role in the development of myocarditis, but the role of T follicular helper (Tfh) cells in the development of autoimmune myocarditis has not previously been reported. Here, we investigated this involvement by using a rat model of experimental autoimmune myocarditis (EAM). Inflammatory cell infiltration, myocardial structure destruction and tissue necrosis were observed in EAM myocardial tissues, and the percentages of CD4^+^CXCR5^+^ Tfh cells and CD19^+^ B cells were both significantly higher in spleen and myocardial tissues of the EAM model as compared with the control group. Furthermore, the expression levels of interleukin‐21, CXCL13 and myosin antibody were significantly higher in the serum of rats with EAM compared with the control group on days 14 and 35 after immunization. Fourteen or 35 days after immunization, the expression levels of interleukin‐21 and CXCL13 were both significantly higher in myocardial tissues of rats with EAM as compared with the control group. Our findings suggest that Tfh cell balance is disrupted during the pathological process of autoimmune myocarditis.

## Graphical abstract

We constructed a model of experimental autoimmune myocarditis, which was used to show that T follicular helper cells can migrate to nonlymphoid tissues. The expression levels of interleukin‐21 and CXCL13 were elevated in serum and spleen of rats with autoimmune myocarditis. T follicular helper cells might play a key role in the pathogenesis of autoimmune myocarditis, and this may provide a basis for identification of new therapeutic targets.

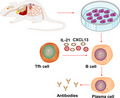

**Abbreviations**
CCK‐8: Cell Counting Kit 8
EAM: experimental autoimmune myocarditis
H&E: hematoxylin and eosin
IL‐21: interleukin‐21
IL‐21R: interleukin‐21 receptor
MYSAb: myosin antibody
qRT‐PCR: quantitative RT‐PCR
Th: T helper
Tfh: T follicular helper

Myocarditis is an inflammatory disease of the myocardium that is associated with immune dysfunction and characterized by myocardial necrosis, fibrosis and inflammatory cell infiltration. It may be idiopathic, infectious or autoimmune and may cause dilated cardiomyopathy [1]. As the gold standard for the diagnosis of myocarditis, endomyocardial biopsy is not commonly used in clinical practice; therefore, the diagnosis of myocarditis is challenging. The prognosis of myocarditis varies greatly depending on the cause [2]. It has been confirmed that the pathological immune response plays an extremely important role in the development of myocarditis [3]. Clinical studies have found that hormones are effective in the treatment of severe myocarditis, suggesting that the body’s abnormal immune response is involved in the occurrence and development of myocarditis [4]. T cell‐mediated immunity has been closely linked to autoimmune myocarditis [5]. Therefore, it is of great significance to explore the pathogenesis of autoimmune myocarditis caused by T cells. Recent studies have found that various CD4^+^ T cell subsets show high plasticity in maintaining myocardial immune homeostasis and regulating disease phenotypes [6]. Therefore, understanding the specific role of these T cell subsets may be critical to the development of successful treatment strategies for autoimmune myocarditis.

It is well‐known that T helper (Th) cells can be classified into various subpopulations depending on the functions and the cytokines secreted by them [7, 8, 9]. For a long time, Th2 cells have been considered to be the major T cell subset of helper B cells [10, 11, 12]. Recent studies have found that T follicular helper (Tfh) cells, a T cell subset that assists B cells to produce antibodies, are the basis for the formation of body germinal centers (GCs) and humoral immunity [13, 14, 15]. This subpopulation is located in the lymphoid follicular T cell region of the lymphoid nodule, and the surface induces the expression of the chemokine receptor CXCR5, which migrates to lymphoid follicle B cells under the recruitment of the chemokine CXCR13 secreted by B cells in lymphoid follicles [16, 17]. In recent years, a small number of studies have reported that Tfh cells are involved in the pathogenesis of autoimmune diseases such as systemic lupus erythematosus, rheumatoid arthritis and Sjögren syndrome [18, 19, 20]. Previous studies have demonstrated that Tfh cells help B cells produce abnormally high affinity antibodies [21]. The formation of ectopic GC was found in the secondary lymphoid organs of the lupus mouse model [22]. Tfh cells play a key role in the activation of these ectopic GCs, anti‐dsDNA Ig secretion and organ damage; however, down‐regulation of Tfh cells can reduce antibody production and lupus symptoms. Another study found that interleukin‐21 (IL‐21) was significantly elevated in the labial glands, salivary glands and serum of patients with primary Sjögren syndrome [23]. Similarly, IL‐21 has been shown to play an important role in autoimmune diseases in the lupus mouse model, the rheumatoid arthritis model, the type 1 diabetes model and the glomerulonephritis model [24, 25, 26, 27]. Moreover, in rats with IL‐21 overexpression, the number of plasma cells and B cells undergoing class switching was significantly increased, whereas in rats with depletion of IL‐21 or its receptor, IL‐21R, the synthetic function of T cell‐dependent antibodies was severely impaired [28]. By blocking the interaction between Tfh and B cells, the disease progression of models such as for systemic lupus erythematosus and rheumatoid arthritis can be significantly improved [19, 29]. In an *in vitro* experiment of Tfh B cells, the addition of IL‐21R antibody significantly reduced the amount of immunoglobulin produced by B cells [30]. Past studies have suggested that Th1/Th2 cell imbalance plays an important role in the occurrence and development of myocarditis [31, 32]. However, to date, the role of Tfh cells in the development of autoimmune myocarditis has not been reported.

In view of the key supporting role of Tfh cells in the production of B cell antibodies in autoimmune diseases, our study aimed to explore the role of Tfh cells in experimental autoimmune myocarditis (EAM) from rats with autoimmune myocarditis.

## Materials and methods

### Preparation of porcine cardiac myosin

The porcine cardiac myosin stock at a concentration of 11.6 mg·mL^−1^ was diluted to a 10‐mg·mL^−1^ solution by sterile PBS buffer. An equal volume of porcine cardiac myosin solution (1 mg·mL^−1^) and Freund’s complete adjuvant (containing mycobacteria, 10 mg·mL^−1^; F5881; Sigma, Shanghai, China) were separately extracted with two 5‐mL glass syringes. Subsequently, the porcine cardiac myosin was fully emulsified. To identify whether the porcine cardiac myosin was completely emulsified, we dripped a drop of the emulsion into the ice water. If not dispersed, it was completely emulsified on the surface of the water. If immediately dispersed, it was not emulsified sufficiently. The emulsification process was performed in the dark and in sterile conditions. After the emulsification was completed, the concentration of porcine cardiac myosin was 0.5 mg·mL^−1^.

### EAM model

Ten female Lewis rats were randomly divided into the EAM model group (*n* = 8) and control group (*n* = 2). In the EAM model group, 0.2 mL porcine cardiac myosin and Freund’s complete adjuvant mixed milk (containing 0.1 mg porcine cardiac myosin) were injected subcutaneously into the left groin and underarm. After 7 days, the EAM model rats were injected at the same dose in the right groin and underarm again. The control group was subcutaneously injected with the same dose of PBS instead of porcine cardiac myosin. The experiments were performed in accordance with the *Guide for the Care and Use of Laboratory Animals* of the National Institutes of Health. Our research was approved by the Ethics Committee of Zhejiang Provincial People’s Hospital.

### Specimen collection

Blood was collected from the orbit of the rats on the 14th and 35th days, respectively. After the rats were sacrificed, the spleen and heart were removed under aseptic conditions. According to the vertical axis of the interventricular septum, the heart was divided into two parts: one half was fixed with 10% neutral formaldehyde for histopathological study; and the other half was placed in the cryotube, frozen in liquid nitrogen at −196 °C. After 24 h, it was stored in a refrigerator at −80 °C for molecular biology research.

### Hematoxylin and eosin staining

Fresh heart tissues were fixed in 4% paraformaldehyde for more than 24 h. After removing the tissues from the fixative, the tissues were smoothed with a scalpel in a fume hood. The trimmed tissues were dehydrated through a series of alcohol (Sinopharm Chemical Reagent Co., Ltd., Beijing, China) in sequence. The wax‐impregnated tissues were embedded. The sections were sliced to a thickness of 4 μm and were placed in a 60 °C oven. Paraffin sections were dewaxed to water. The sections were stained with Harris hematoxylin for 5–10 min, followed by eosin staining for 1–3 min. After dehydration, histopathological changes were observed under a microscope (Olympus, Hatagaya, Japan). Myopathological scores were calculated using a semiquantitative analysis of Rezkalla. Five fields were randomly taken from each section, and the ratio of the area of inflammatory cell infiltration and necrotic area to the entire field of view in each field of view was calculated. Scoring criteria were as follows: no inflammatory cell infiltration (0 points), inflammatory cell infiltration <5% (1 point), inflammatory cell infiltration 5–10% (2 points), inflammatory cell infiltration 10–20% (3 points) and inflammatory cell infiltration >20% (4 points).

### Flow cytometry assay

After the rats were sacrificed, spleen tissues and myocardial tissues were removed and placed in precooled PBS. After that, the tissues were placed on a 200 mesh screen, gently grounded with a syringe stopper and rinsed with a 5‐mL lymphocyte separation solution. The lymphocyte separation with the cell suspension was added into a clean 15‐mL tube. On the upper layer of the cell suspension, 2 mL serum‐free 1640 was gently superimposed, followed by centrifugation at 800 ***g*** for 30 min at room temperature. The middle layer of white mistlike lymphocytes was pipetted. After that, collected cells were incubated with 100 µL Fc receptor blocker (anti‐CD16/32 Ig; 1 : 200) at 4 °C for 30 min, followed by centrifugation. After discarding the supernatant, the cell pellet was retained, and the PBS was resuspended in two portions for two staining protocols. One portion was incubated with 100 µL anti‐CXCR5 (1 : 50) at 37 °C for 2 h, followed by incubation with R‐Phycoerythrin (1 : 500), CD4‐FITC (1 : 100) and 7‐aminoactinomycin D (1 : 50) at room temperature in the dark for 1 h. The other portion was incubated with 10 µL 7‐aminoactinomycin D (1 : 50) and 5 µL CD19‐FITC (1 : 100) at 4 °C for 30 min in the dark. After staining, the suspension was stored at 4 °C in the dark overnight. CD4^+^CXCR5^+^ Tfh and B cell ratios were examined with flow cytometry.

### ELISA

Blood was collected from the orbit of the rats on the 14th and 35th days for ELISA. According to the manufacturer’s instructions, myosin antibody (MYSAb), CXCL13 and IL‐21 were detected using Rat MYSAb ELISA Kit (OM626374; OmnimAbs, New Jersey, USA), Rat CXCL13 ELISA Kit (SEB601Ra; Cloud‐clone, China) and Rat ELISA Kit (SEB688Ra; Cloud‐clone, Hangzhou, China), respectively.

### Quantitative RT‐PCR

TaKaRa MiniBEST Universal RNA Extraction Kit (Catalog #9767; Takara, Dongjing, Japan) was used to extract RNA from myocardial tissues according to the manufacturer’s instructions. To assess RNA quality, we determined the *A*_260_/*A*_280_ ratio. The ratio met the experimental requirements between 1.8 and 2.0. After that, the total RNA was stored in a −80 °C freezer. The total RNA was reverse transcribed into cDNA under the following conditions: 37 °C for 15 min; 85 °C for 5 s and 4 °C hold. Then the expression levels of target genes were detected using quantitative RT‐PCR (qRT‐PCR) under the following thermocycler conditions: 95 °C for 5 min, 40 cycles of 95 °C for 20 s and 62 °C for 15 s, followed by 72 °C for 3 min. The primer information for IL‐21 and CXCL13 is listed in Table 1. GAPDH was used as an internal control. The relative expression levels were calculated with the $2^{-\Delta \Delta C_{t}}$ method.

**Table 1:** Primer information for qRT‐PCR.

Gene name	5′–3′ sequence	Product size (bp)
*GAPDH*	5′‐CAAGTTCAACGGCACAGTCAAG‐3’ (forward)	123
	5′‐ACATACTCAGCACCAGCATCAC‐3’ (reverse)	
*IL‐21*	5′‐GGACCGTGGCCCATAAATCA‐3’ (forward)	166
	5′‐GCAAAAGCTTCGTGCTCACA‐3’ (reverse)	
*CXCL13*	5′‐AAGCCACTGTCACCCCAAAA‐3’ (forward)	108
	5′‐ACAGCCGTGTTTGTAGAGGG‐3’ (reverse)	

### Tfh/B cell coculture assays

CD19^+^ B cells and each group of Tfh cells (including Tfh1, Tfh2 and Tfh17) were purified from blood on the 14th and 35th days after immunization. The levels of IL‐21 and CXCL13 in supernatant of Tfh1, Tfh2 and Tfh17 were measured using ELISAs. Prior to coculture, the purity of B cells and each group of Tfh cells was >95%. An equal number of purified Tfh1, Tfh2, Tfh17 and CD19^+^ B cells (1 × 10^5^ cells/well) were cocultured in a 96‐well plate and then stimulated with staphylococcal enterotoxin B (Sigma) for 6 days. Cell Counting Kit 8 (CCK‐8) assay was performed to detect CD19^+^ B cell viability. After purification, B cells were incubated in a 96‐well plate (2000 cells per well). Ten microlitres CCK‐8 reagent was added into each well. After 24 h, the cell viability was measured at a 450‐nm wavelength using a microplate reader (Labsystem, Shanghai, China).

### Statistical analysis

graphpad prism 7.0 (GraphPad, San Diego, CA, USA) was used for statistical analyses. All experiments were independently repeated at least three times. The data are expressed as mean ± SD. Comparisons between two groups were analyzed with unpaired *t*‐test, whereas one‐way ANOVA was presented for comparison between multiple groups. *P*‐value <0.05 was considered statistically significant.

## Results

### Increased myocardial inflammation in myocardial tissues of the EAM model

**Fig. 1:**
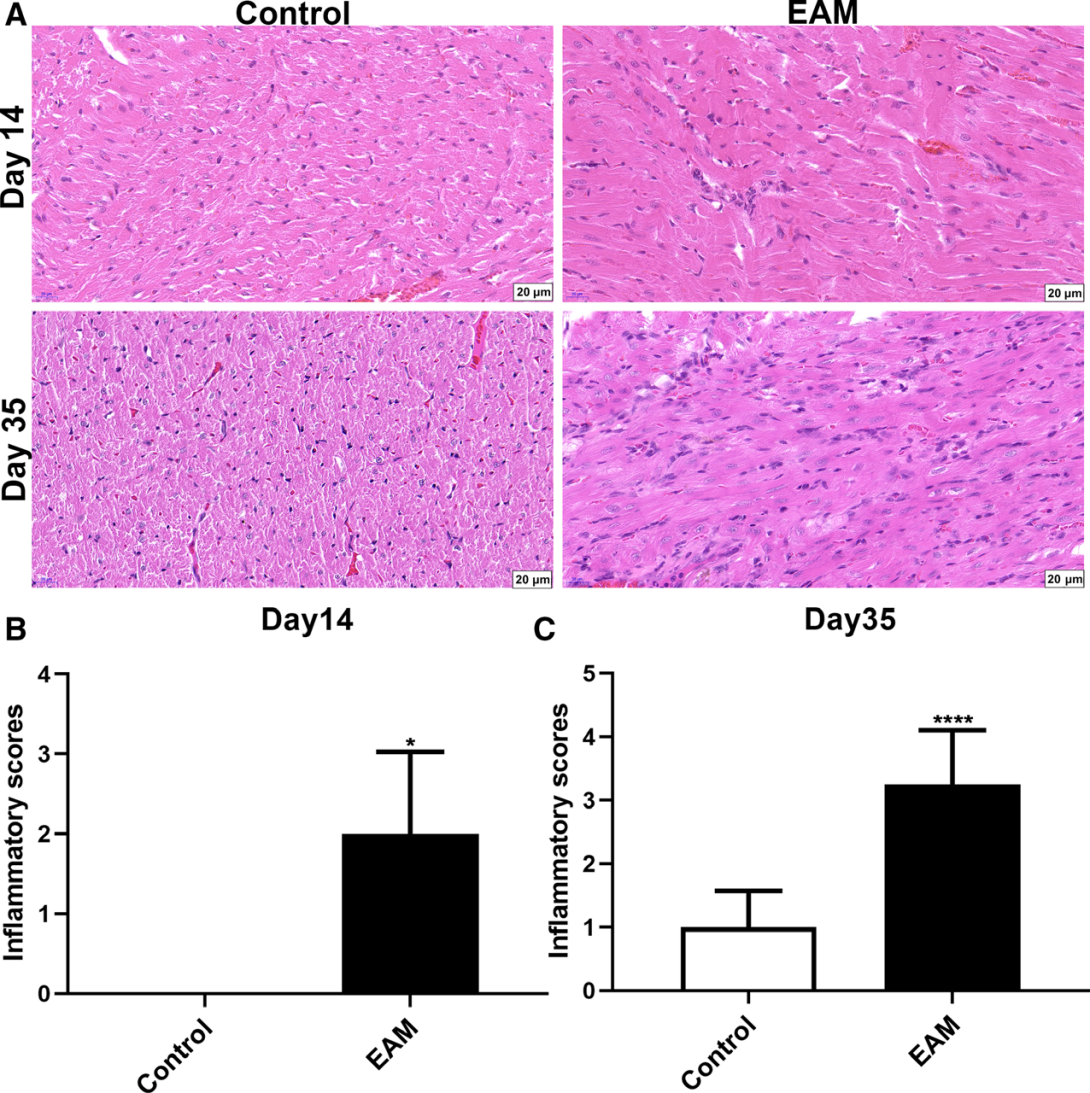
Histopathology analysis. H&E staining of myocardial tissues was observed 14 or 35 days after the initial immunization. (A) Representative images of H&E staining of myocardial tissues. Scale bars: 20 μm; original magnification: ×200. (B, C) H&E staining inflammatory scores on day 14 or 35 after immunization. *P < 0.05, ****P < 0.0001. The data are expressed as mean ± SD; each experiment was *n* ≥ 3. Comparisons between two groups were analyzed with unpaired *t*‐test.

Hematoxylin and eosin (H&E) staining results showed that inflammatory cell infiltration, myocardial structure destruction and tissue necrosis were observed in the myocardial tissues of the EAM model group (Fig. 1A). In addition, myocardial tissue damage 35 days after the initial immunization was significantly more severe than 14 days after the initial immunization (Fig. 1A). There was a large number of focal inflammatory cell infiltrates, mainly lymphocytes, accompanied by capillary expansion and myocardial cell necrosis in the myocardial tissues of the EAM model group 35 days after the initial immunization. Inflammatory scores were significantly higher in the EAM model group 14 or 35 days after the initial immunization compared with those in the control group (Fig. 1B,C).

### **Increased percentage of the CD4** ^+^ **CXCR5^+^ Tfh cells in spleen and myocardial tissues of the EAM model**

**Fig. 2:**
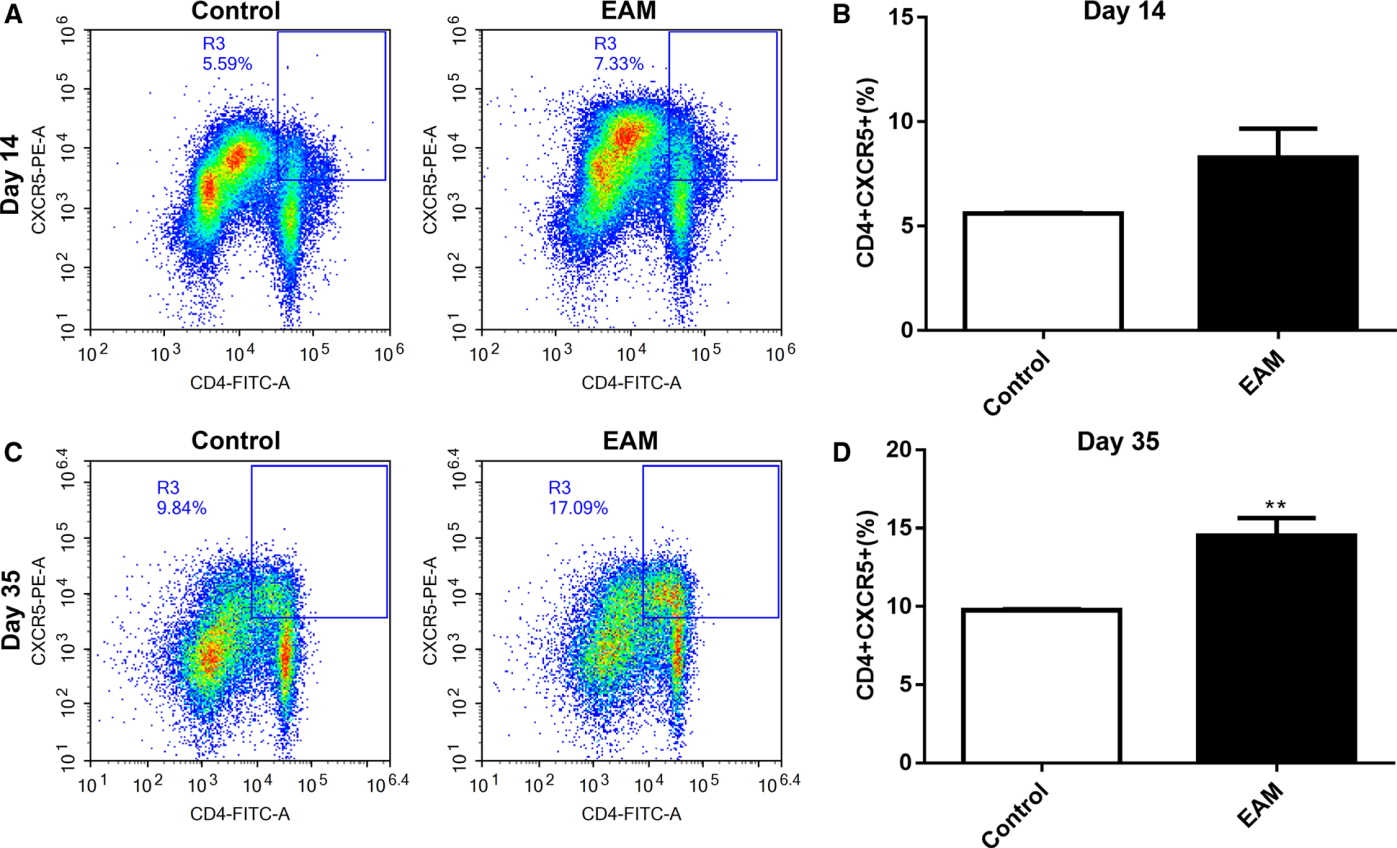
Increased percentage of the CD4^+^CXCR5^+^ Tfh cells in spleen tissues of the EAM model. (A, B) The percentage of CD4^+^CXCR5^+^ Tfh cells in spleen tissues of the EAM model on day 14 after immunization. (C, D) The percentage of CD4^+^CXCR5^+^ Tfh cells in spleen tissues of the EAM model on day 35 after immunization. **P < 0.01. The data are expressed as mean ± SD; each experiment was *n* ≥ 3. Comparisons between two groups were analyzed with unpaired *t*‐test.

**Fig. 3:**
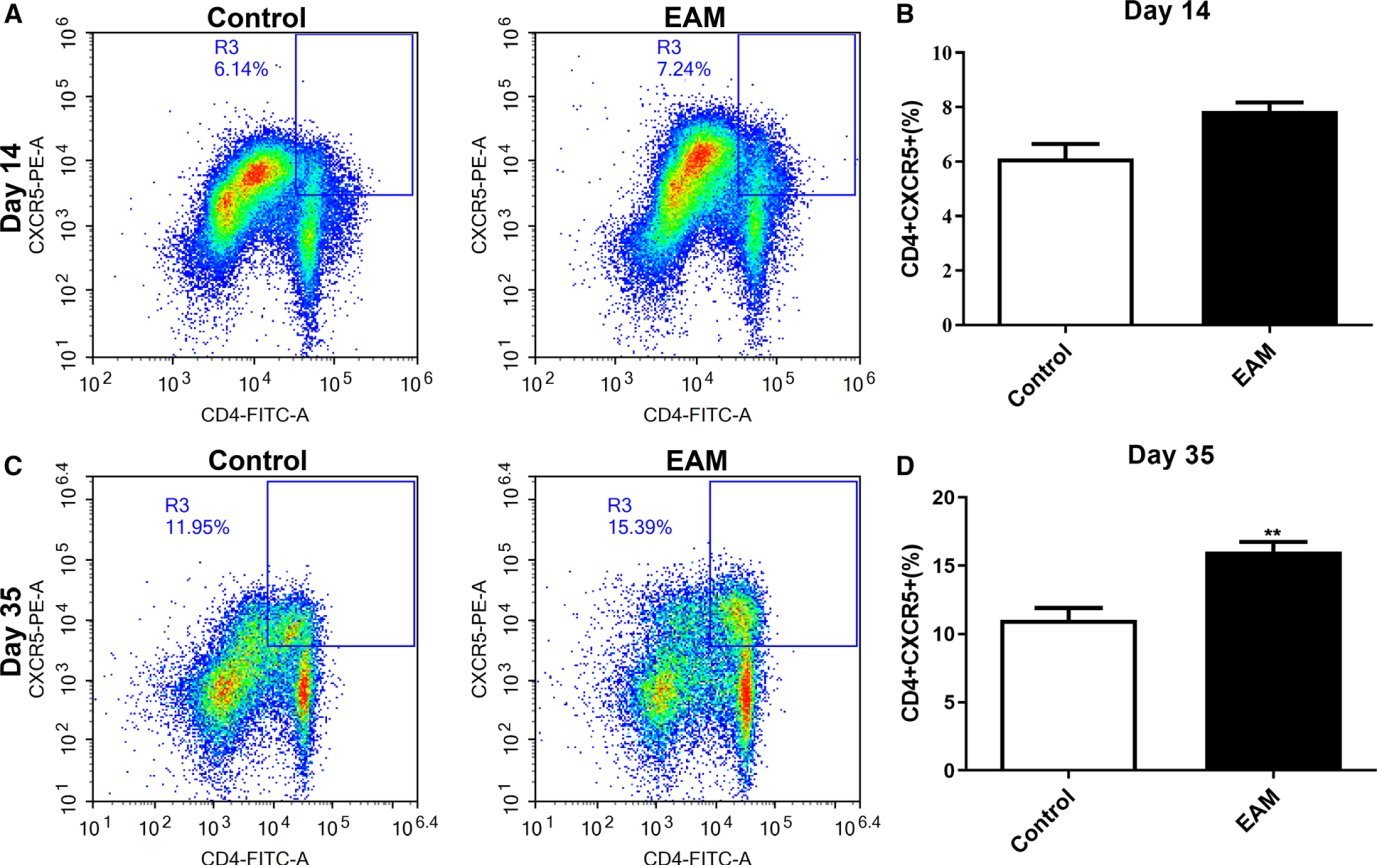
Increased percentage of CD4^+^CXCR5^+^ Tfh cells in myocardial tissues of the EAM model. (A, B) The percentage of CD4^+^CXCR5^+^ Tfh cells in myocardial tissues of the EAM model on day 14 after immunization. (C, D) The percentage of CD4^+^CXCR5^+^ Tfh cells in myocardial tissues of the EAM model on day 35 after immunization. **P < 0.01. The data are expressed as mean ± SD; each experiment was *n* ≥ 3. Comparisons between two groups were analyzed with unpaired *t*‐test.

The percentage of CD4^+^CXCR5^+^ Tfh cells in spleen and myocardial tissues of the EAM model was detected by flow cytometry analysis. We found that there was no statistically significant difference on day 14 after immunization in rat spleen tissues between two groups (Fig. 2A,B). However, 35 days after immunization, the results showed that the percentage of CD4^+^CXCR5^+^ Tfh cells in spleen tissues of the EAM model group was significantly higher than in the control group (Fig. 2C,D). Similar results were observed in myocardial tissues. As shown in Fig. 3A,B, no significant difference in CD4^+^CXCR5^+^ Tfh cells on day 14 after immunization in rat myocardial tissues was found between the EAM model and control groups. Thirty‐five days after immunization, CD4^+^CXCR5^+^ Tfh cells had a significantly higher percentage in rat myocardial tissues of the EAM model group than the control group (Fig. 3C,D).

### **Increased percentage of CD19** ^+^ **B cells in spleen tissues of the EAM model**

**Fig. 4:**
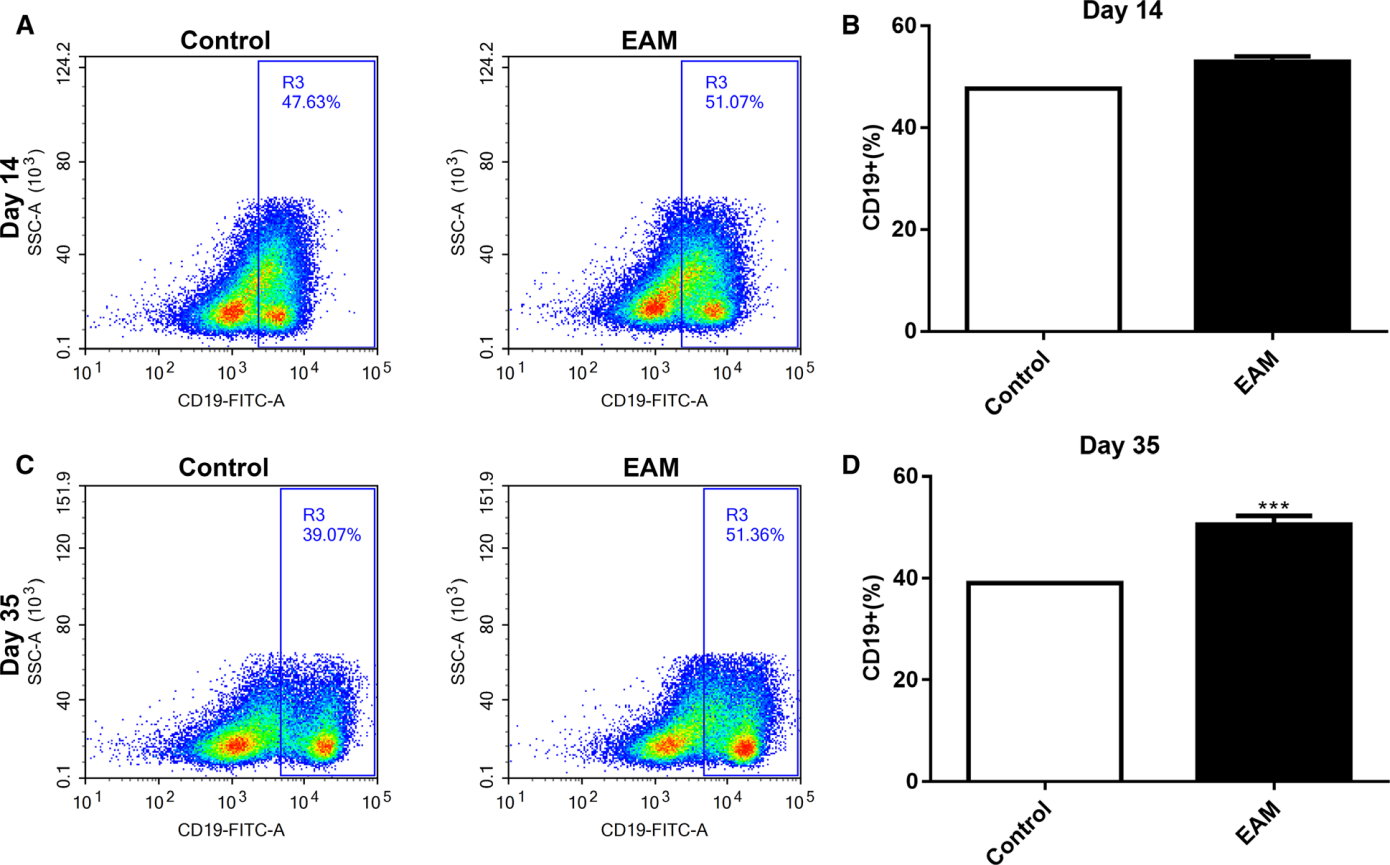
Increased percentage of the CD19^+^ B cells in spleen tissues of the EAM model. (A, B) The percentage of CD19^+^ B cells in spleen tissues of the EAM model on day 14 after immunization. (C, D) The percentage of CD19^+^ B cells in spleen tissues of the EAM model on day 35 after immunization. ***P < 0.001. The data are expressed as mean ± SD; each experiment was *n* ≥ 3. Comparisons between two groups were analyzed with unpaired *t*‐test.

We detected the percentage of CD19^+^ B cells in spleen tissues of the EAM model using flow cytometry analysis. The results showed that, 14 days after immunization, the percentage of CD19^+^ B cells in spleen tissues of the EAM model was higher than in the control group; however, it was not statistically significant (Fig. 4A,B). Thirty‐five days after immunization, we found that the percentage of CD19^+^ B cells in spleen tissues of the EAM model group was significantly higher than in the control group (Fig. 4C,D).

### Elevated expression levels of IL‐21, CXCL13 and MYSAb in the serum of rats with EAM

**Fig. 5:**
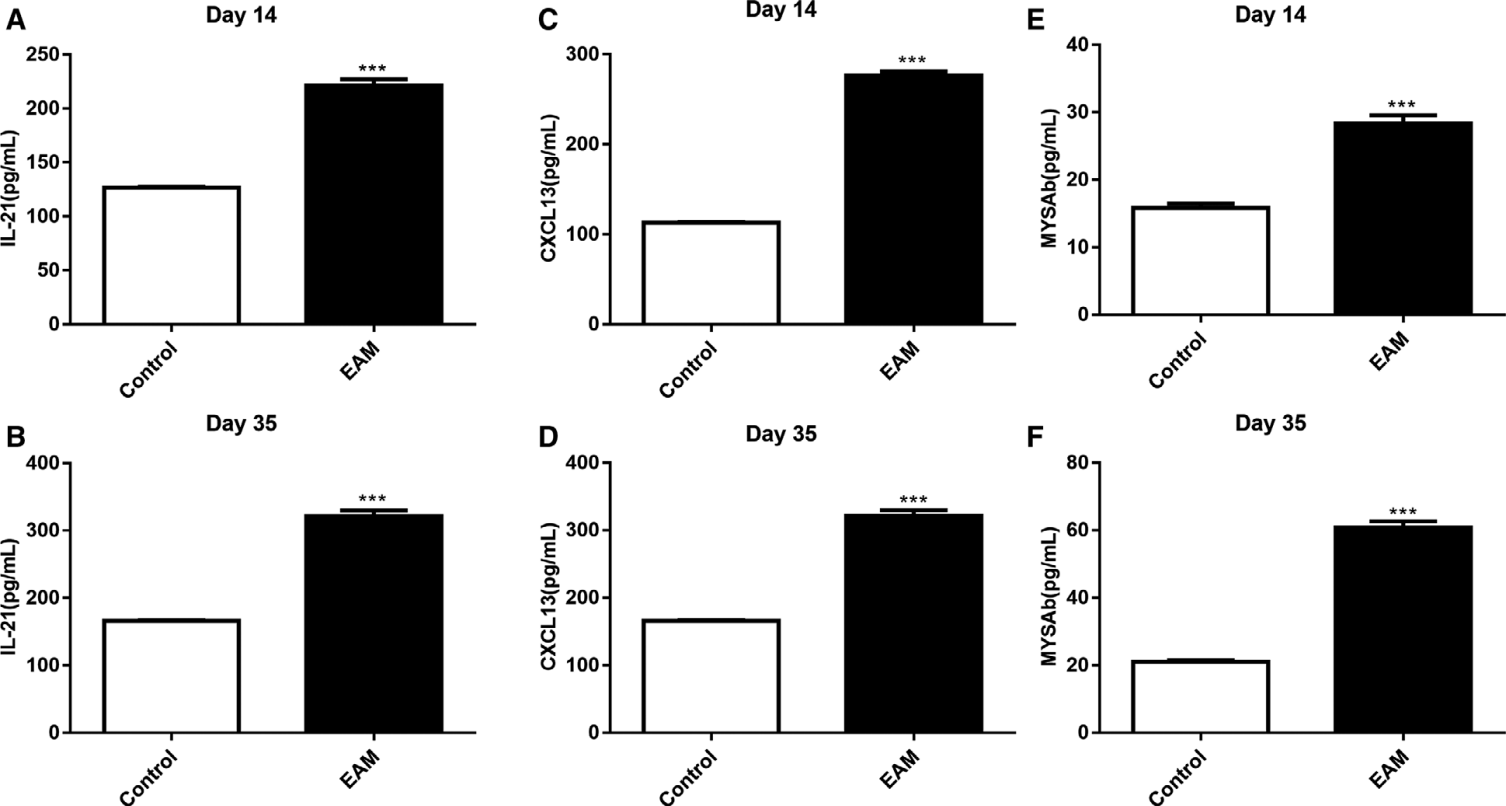
Elevated expression levels of IL‐21, CXCL13 and MYSAb in the serum of rats with EAM. (A, B) ELISA results showing the expression level of IL‐21 in the serum of rats with EAM on day 14 or 35 after immunization. (C, D) The expression level of CXCL13 in the serum of rats with EAM on day 14 or 35 after immunization using ELISA. (E, F) The expression level of MYSAb in the serum of rats with EAM on day 14 or 35 after immunization using ELISA. ***P < 0.001. The data are expressed as mean ± SD; each experiment was *n* ≥ 3. Comparisons between two groups were analyzed with unpaired *t*‐test.

The expression levels of IL‐21, CXCL13 and MYSAb in the serum of rats with EAM were detected using ELISA. On day 14 or 35 after immunization, we found that the expression level of IL‐21 was significantly elevated in the serum of rats with EAM compared with the control group (Fig. 5A,B). Furthermore, the results showed that the expression level of CXCL13 was significantly higher in the serum of rats with EAM compared with the control group (Fig. 5C,D). As for MYSAb, our results showed that its expression level was significantly higher in the serum of rats with EAM compared with the control group (Fig. 5E,F).

### Elevated expression levels of IL‐21 and CXCL13 in myocardial tissues of rats with EAM

**Fig. 6:**
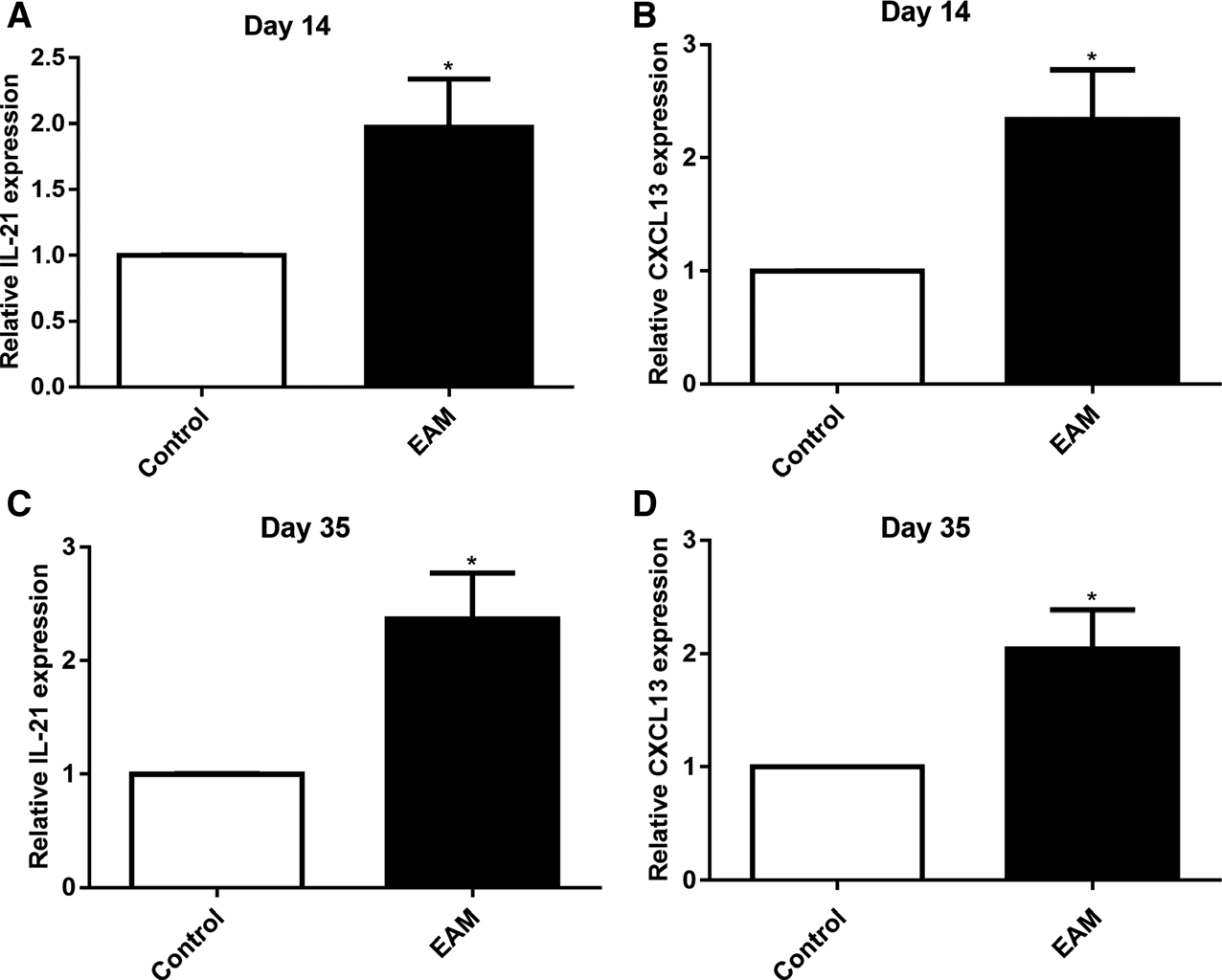
Elevated expression levels of IL‐21 and CXCL13 in myocardial tissues of rats with EAM. (A, B) qRT‐PCR results showing the expression levels of IL‐21 and CXCL13 in myocardial tissues of rats with EAM on day 14 after immunization. (C, D) qRT‐PCR results showing the expression levels of IL‐21 and CXCL13 in myocardial tissues of rats with EAM on day 35 after immunization. *P < 0.05. The data are expressed as mean ± SD; each experiment was *n* ≥ 3. Comparisons between two groups were analyzed with unpaired *t*‐test.

We examined the expression levels of IL‐21 and CXCL13 in myocardial tissues of rats with EAM using qRT‐PCR. As shown in Fig. 6A,B, 14 days after immunization, the expression levels of IL‐21 and CXCL13 were both significantly higher in myocardial tissues of rats with EAM compared with the control group. Moreover, we found that the expression levels of IL‐21 and CXCL13 were both significantly elevated in myocardial tissues of rats with EAM compared with the control group on day 35 after immunization (Fig. 6C,D).

### Tfh cells in the serum of rats with EAM can produce increased levels of IL‐21 and CXCL13

**Fig. 7:**
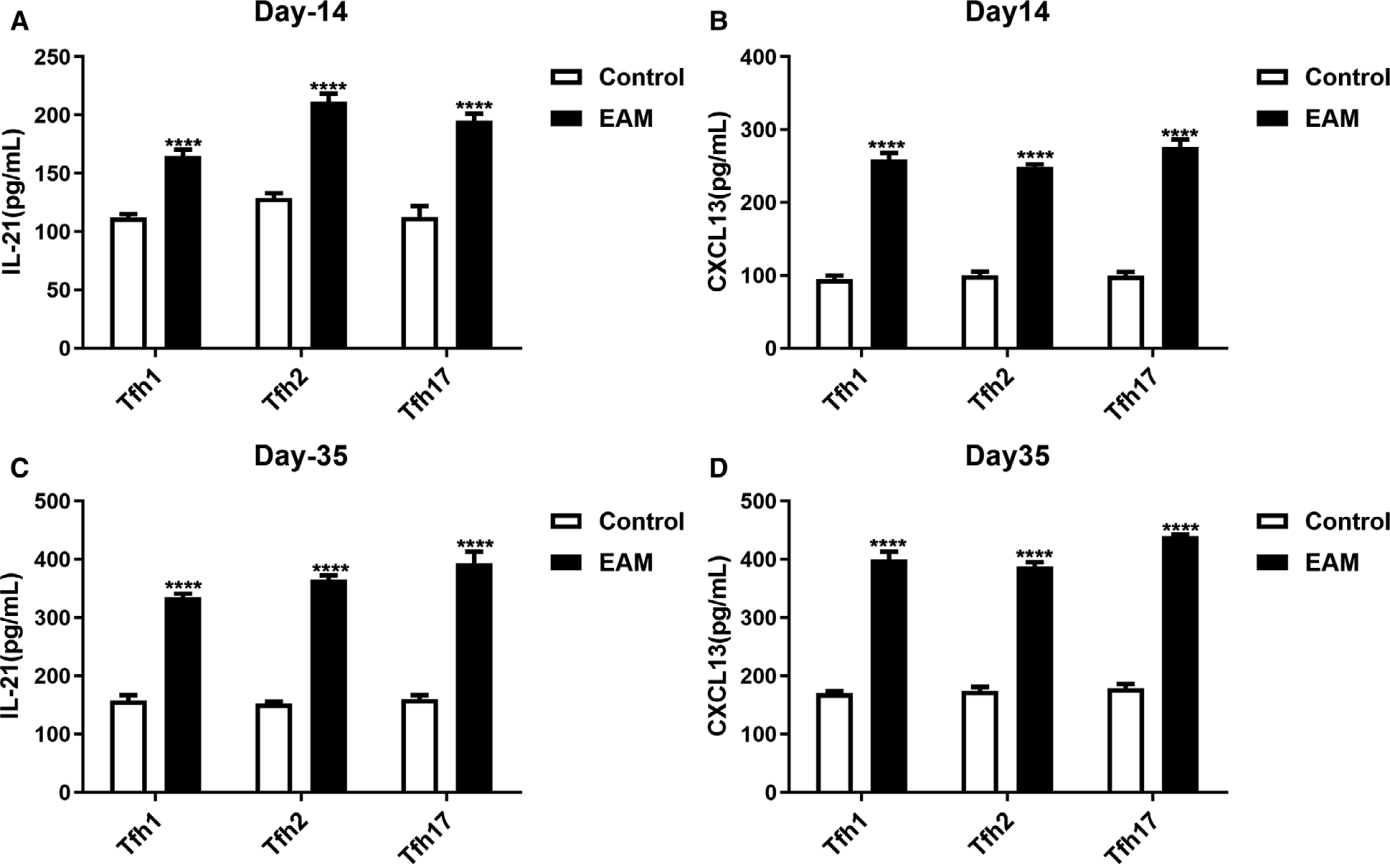
Tfh cells in the serum of rats with EAM can produce increased levels of IL‐21 and CXCL13. (A, B) ELISA results showing the expression level of IL‐21 and CXCL13 in Tfh cells on day 14 after immunization. (C, D) ELISA results showing the expression level of IL‐21 and CXCL13 in Tfh cells on day 35 after immunization. ****P < 0.0001. The data are expressed as mean ± SD; each experiment was *n* ≥ 3. Comparisons between two groups were analyzed with one‐way ANOVA.

After the purification of each group of Tfh cells in the serum of rats with EAM on day 14 or 35 after immunization, ELISA was performed to examine the levels of IL‐21 and CXCL13 in the supernatant. On day 14 after immunization, the levels of IL‐21 (Fig. 7A) and CXCL13 (Fig. 7B) in each group of Tfh cells in the serum of rats with EAM were significantly higher than those in the control group. Furthermore, when Tfh cells were immunized on day 35, higher levels of IL‐21 (Fig. 7C) and CXCL13 (Fig. 7D) were found in the EAM group compared with the control group.

### Tfh cells in the serum of rats with EAM promote B cell viability *in vitro*

**Fig. 8:**
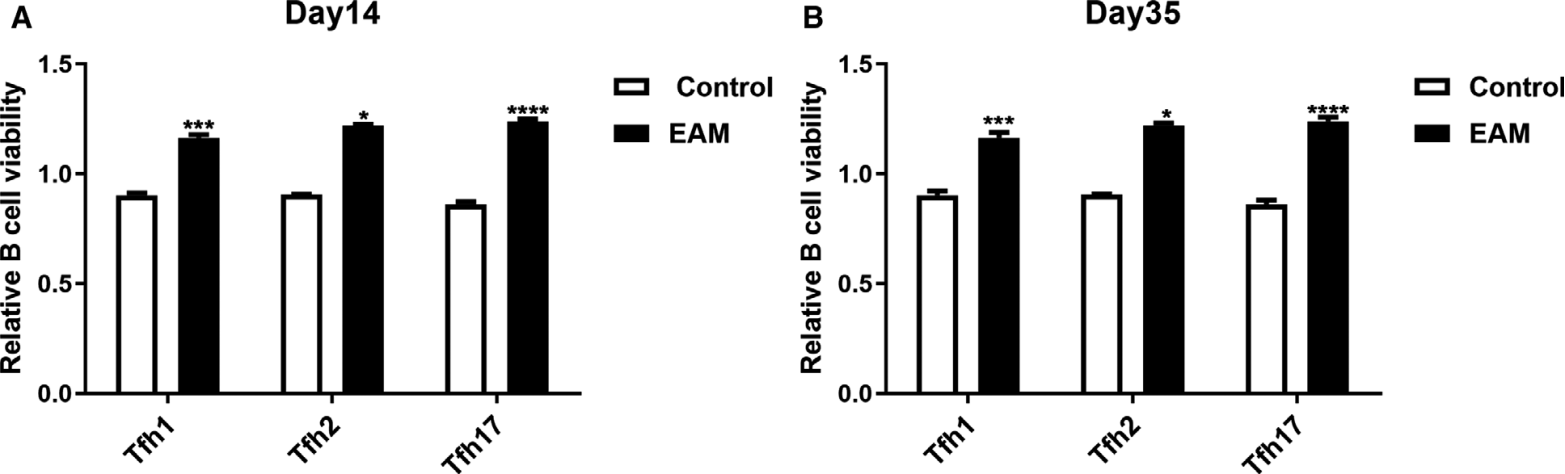
Tfh cells in the serum of rats with EAM promote B cell viability *in vitro* on day 14 (A) or 35 (B) after immunization. *P < 0.05, ***P < 0.001, ****P < 0.0001. The data are expressed as mean ± SD; each experiment was *n* ≥ 3. Comparisons between two groups were analyzed with one‐way ANOVA.

After Tfh/B cells coculture, CCK‐8 was used to detect B cell viability. The results showed that, compared with the control group, Tfh cells, including Tfh1, Tfh2 and Tfh17, in the serum of rats with EAM on day 14 or 35 after immunization both significantly promoted B cell viability *in vitro* (Fig. 8A,B).

## Discussion

In this study, we constructed the EAM model. Based on the characteristics of Tfh cells that can migrate to nonlymphoid tissues and form lymphoid tissues containing ectopic GCs, we investigated the overexpression of Tfh and B cells in spleen and myocardial tissues of rats with autoimmune myocarditis. The expression levels of IL‐21, CXCL13 and MYSAb were elevated in the serum of rats with EAM. Furthermore, we also observed the expression of cytokine IL‐21 and chemokine CXCL13 in the spleen of rats with autoimmune myocarditis. *In vitro*, Tfh cells (including Tfh1, Tfh2 and Tfh17) in the serum of rats with EAM can produce increased levels of IL‐21 and CXCL13. Furthermore, Tfh1, Tfh2 and Tfh17 cells in the serum of rats with EAM promote B cell viability. Our study revealed that Tfh cells might play a key role in the pathogenesis of autoimmune myocarditis.

Tfh cells are a group of independent T cell subsets, which are significantly different from Th1, Th2, Th17 and regulatory T cells. It has been well recognized that follicular homing receptor CXCR5 is an important molecular marker of Tfh cells [33]. Its ligand is the chemokine CXCL13 and is mainly secreted by follicular dendritic cells [34]. After receiving the body stimulation signal, the Tfh cells inducibly express CXCR5 and underexpress the T cell homing receptor CCR7 [35]. Therefore, CXCR5 becomes an important ‘transporter molecule’ for Tfh migration and localization, and is also an important surface marker of Tfh cells. In this study, our results showed that the percentage of CD4^+^CXCR5^+^ Tfh cells was significantly higher in spleen and myocardial tissues of the EAM model than the control group 35 days after immunization. Our results indicated that Tfh cells could play an important role in myocarditis.

IL‐21 is a major cytokine in which Tfh cells perform effector functions, which is mainly produced in Tfh cells [36]. A previous study found that rats with IL‐21 knockout had essentially no Tfh cells, and GC also disappeared, indicating that IL‐21 may be required for Tfh cell differentiation [37]. Its receptor, IL‐21R, is mainly expressed on the surface of B cells, and the combination of the two can induce differentiation of all B cell subsets into immunoglobulin‐secreting cells, which is a key factor for stimulating plasma cell differentiation, and T cell‐dependent antigen in B cells. The cytokine IL‐21 can be produced to assist B cells in a humoral immune response [38]. In this study, we found that the percentage of CD19^+^ B cells was significantly higher in spleen tissues of the EAM model than the control group 35 days after immunization. *In vitro*, Tfh cells in the serum of rats with EAM promote B cell viability *in vitro*. On days 14 or 35 after immunization, the expression level of IL‐21 was significantly elevated in the serum of rats with EAM compared with the control group. Furthermore, the expression level of IL‐21 was also significantly higher in myocardial tissues of rats with EAM compared with the control group. Studies have reported that the levels of IL‐21 in the serum of EAM rats were significantly higher than in normal controls [39]. Myocarditis is a common disease in the cardiovascular system. Autoimmunity is the main cause of disease. The abnormality of Tfh cells is closely related to the occurrence and development of other classic autoimmune diseases. Our results indicated that overexpression of Tfh cells (including Tfh1, Tfh2 and Tfh17) in autoimmune myocarditis might lead to the increased secretion of IL‐21, causing autoimmune myocarditis to produce excess autoantibodies.

Studies have found that the B cell chemokine CXCL13 is involved in the infiltration of Tfh cells [34]. Moreover, CXCL13 may enhance humoral immunity via recruiting both Tfh and GC B cells [34, 40]. In our study, we found elevated expression levels of CXCL13 in serum and myocardial tissues of rats with EAM. CXCL13 has been considered as a plasma biomarker of GC activity. In a stable internal environment, Tfh cells are localized in lymphoid follicles, and their regulation of B cell proliferation and plasma cell differentiation is also mainly done in lymphoid follicles, providing necessary assistance for the body to produce normal antibodies. However, when pathological conditions such as autoimmune diseases occur, Tfh cells undergo pathological activation and amplification, including abnormal expression of molecules such as CXCR5, and migration to nonlymphoid tissues of the body to form lymphoid tissues containing ectopic GC. Furthermore, our findings suggested that Tfh cells could generate a large number of cytokines IL‐21 and CXCL13 to stimulate B cells to produce a large number of autoantibodies, which ultimately accelerates the development of autoimmune myocarditis.

Therefore, the abnormality of Tfh cells in autoimmune myocarditis and its regulation mechanism could play a key role in the pathogenesis of autoimmune myocarditis.

## Conclusions

In this study, we successfully constructed the EAM model. We found that Tfh cells can migrate to nonlymphoid tissues. The expression levels of IL‐21 and CXCL13 were elevated in the serum and spleen of rats with autoimmune myocarditis. Our study revealed that Tfh cells might play a key role in the pathogenesis of autoimmune myocarditis, which provides a basis for finding new therapeutic targets.

## Conflict of interest

The authors declare no conflict of interest.

## Author contributions

HS conceived and designed the study. QX conducted most of the experiments and data analysis, and wrote the manuscript. YM and LW participated in collecting data and helped to draft the manuscript. All authors reviewed and approved the manuscript.

## Data Availability

The datasets analyzed during this study are available from the corresponding author on reasonable request.

## References

[1] Caforio AL , Marcolongo R , Jahns R , Fu M , Felix SB and Iliceto S (2013) Immune‐mediated and autoimmune myocarditis: clinical presentation, diagnosis and management. Heart Failure Rev 18, 715–732. 10.1007/s10741-012-9364-5 23114995

[2] Sinagra G , Anzini M , Pereira NL , Bussani R , Finocchiaro G , Bartunek J and Merlo M (2016) Myocarditis in clinical practice. Mayo Clinic Proc 91, 1256–1266. 10.1016/j.mayocp.2016.05.013 27489051

[3] Epelman S , Liu PP and Mann DL (2015) Role of innate and adaptive immune mechanisms in cardiac injury and repair. Nat Rev Immunol 15, 117–129. 25614321 10.1038/nri3800 PMC4669103

[4] Mahmood SS , Fradley MG , Cohen JV , Nohria A , Reynolds KL , Heinzerling LM , Sullivan RJ , Damrongwatanasuk R , Chen CL , Gupta D *et al* (2018) Myocarditis in patients treated with immune checkpoint inhibitors. J Am Coll Cardiol 71, 1755–1764. 29567210 10.1016/j.jacc.2018.02.037 PMC6196725

[5] Stephenson E , Savvatis K , Mohiddin SA and Marelli‐Berg FM (2017) T‐cell immunity in myocardial inflammation: pathogenic role and therapeutic manipulation. Br J Pharmacol 174, 3914–3925. 27590129 10.1111/bph.13613 PMC5659997

[6] Vdovenko D and Eriksson U (2018) Regulatory role of CD4(+) T cells in myocarditis. J Immunol Res 2018, 4396351. 30035131 10.1155/2018/4396351 PMC6032977

[7] De Feo D , Merlini A , Brambilla E , Ottoboni L , Laterza C , Menon R , Srinivasan S , Farina C , Garcia Manteiga JM , Butti E *et al* (2017) Neural precursor cell‐secreted TGF‐beta2 redirects inflammatory monocyte‐derived cells in CNS autoimmunity. J Clin Investig 127, 3937–3953. 28945200 10.1172/JCI92387 PMC5663358

[8] Moss RB , Moll T , El‐Kalay M , Kohne C , Soo Hoo W , Encinas J and Carlo DJ (2004) Th1/Th2 cells in inflammatory disease states: therapeutic implications. Exp Opini Biol Ther 4, 1887–1896. 10.1517/14712598.4.12.1887 15571451

[9] Zimmermann J , Kuhl AA , Weber M , Grun JR , Loffler J , Haftmann C , Riedel R , Maschmeyer P , Lehmann K , Westendorf K *et al* (2016) T‐bet expression by Th cells promotes type 1 inflammation but is dispensable for colitis. Mucosal Immunol 9, 1487–1499. 26883725 10.1038/mi.2016.5

[10] Dwyer DF , Woodruff MC , Carroll MC , Austen KF and Gurish MF (1950) (2014) B cells regulate CD4+ T cell responses to papain following B cell receptor‐independent papain uptake. J Immunol 193, 529–539. 10.4049/jimmunol.1303247 PMC4203309 24928991

[11] Ellis JS , Guloglu FB and Zaghouani H (2016) Presentation of high antigen‐dose by splenic B220(lo) B cells fosters a feedback loop between T helper type 2 memory and antibody isotype switching. Immunology 147, 464–475. 26749165 10.1111/imm.12579 PMC4799881

[12] Zhu J , Xu Y , Zhu C , Zhao J , Meng X , Chen S , Wang T , Li X , Zhang L , Lu C *et al* (2017) IL‐33 induces both regulatory B cells and regulatory T cells in dextran sulfate sodium‐induced colitis. Int Immunopharmacol 46, 38–47. 28258042 10.1016/j.intimp.2017.02.006

[13] Crotty S (2011) Follicular helper CD4 T cells (TFH). Annu Rev Immunol 29, 621–663. 21314428 10.1146/annurev-immunol-031210-101400

[14] Kubo M (2017) T follicular helper and TH2 cells in allergic responses. Allergol Int 66, 377–381. 28499720 10.1016/j.alit.2017.04.006

[15] Pattarini L , Trichot C , Bogiatzi S , Grandclaudon M , Meller S , Keuylian Z , Durand M , Volpe E , Madonna S , Cavani A *et al* (2017) TSLP‐activated dendritic cells induce human T follicular helper cell differentiation through OX40‐ligand. J Exp Med 214, 1529–1546. 28428203 10.1084/jem.20150402 PMC5413322

[16] Havenith SH , Remmerswaal EB , Idu MM and van Donselaar‐van der Pant KA , van der Bom N , Bemelman FJ , van Leeuwen EM , ten Berge IJ and van Lier RA (2014) CXCR5+CD4+ follicular helper T cells accumulate in resting human lymph nodes and have superior B cell helper activity. Int Immunol 26, 183–192. 24291746 10.1093/intimm/dxt058

[17] Morita R , Schmitt N , Bentebibel SE , Ranganathan R , Bourdery L , Zurawski G , Foucat E , Dullaers M , Oh S , Sabzghabaei N *et al* (2011) Human blood CXCR5(+)CD4(+) T cells are counterparts of T follicular cells and contain specific subsets that differentially support antibody secretion. Immunity 34, 108–121. 21215658 10.1016/j.immuni.2010.12.012 PMC3046815

[18] Verstappen GM , Meiners PM , Corneth OBJ , Visser A , Arends S , Abdulahad WH , Hendriks RW , Vissink A , Kroese FGM and Bootsma H (2017) Attenuation of follicular helper T cell‐dependent B cell hyperactivity by Abatacept treatment in primary Sjogren's syndrome. Arthritis Rheumatol 69, 1850–1861. 28564491 10.1002/art.40165

[19] Tang Y , Wang B , Sun X , Li H , Ouyang X , Wei J , Dai B , Zhang Y and Li X (2017) Rheumatoid arthritis fibroblast‐like synoviocytes co‐cultured with PBMC increased peripheral CD4(+) CXCR5(+) ICOS(+) T cell numbers. Clin Exp Immunol 190, 384–393. 28833034 10.1111/cei.13025 PMC5680054

[20] Kim SJ , Lee K and Diamond B (2018) Follicular helper T cells in systemic lupus erythematosus. Front Immunol 9, 1793. 30123218 10.3389/fimmu.2018.01793 PMC6085416

[21] Mazerolles F , Picard C , Kracker S , Fischer A and Durandy A (2013) Blood CD4+CD45RO+CXCR5+ T cells are decreased but partially functional in signal transducer and activator of transcription 3 deficiency. J Allergy Clin Immunol 131, 1146–1156, 1156.e1–5. 23403044 10.1016/j.jaci.2012.12.1519

[22] Bates MA , Akbari P , Gilley KN , Wagner JG , Li N , Kopec AK , Wierenga KA , Jackson‐Humbles D , Brandenberger C , Holian A *et al* (2018) Dietary docosahexaenoic acid prevents silica‐induced development of pulmonary ectopic germinal centers and glomerulonephritis in the lupus‐prone NZBWF1 mouse. Front Immunol 9, 2002. 30258439 10.3389/fimmu.2018.02002 PMC6143671

[23] Kwok SK , Lee J , Yu D , Kang KY , Cho ML , Kim HR , Ju JH , Lee SH , Park SH and Kim HY (2015) A pathogenetic role for IL‐21 in primary Sjogren syndrome. Nat Rev Rheumatol 11, 368–374. 25584898 10.1038/nrrheum.2014.225

[24] Gutierrez T , Mayeux JM , Ortega SB , Karandikar NJ , Li QZ , Rakheja D , Zhou XJ and Satterthwaite AB (2013) IL‐21 promotes the production of anti‐DNA IgG but is dispensable for kidney damage in lyn‐/‐ mice. Eur J Immunol 43, 382–393. 23169140 10.1002/eji.201142095 PMC3768150

[25] Ryden AK , Perdue NR , Pagni PP , Gibson CB , Ratliff SS , Kirk RK , Friesen TJ , Haase C , Coppieters K , von Herrath MG *et al* (2017) Anti‐IL‐21 monoclonal antibody combined with liraglutide effectively reverses established hyperglycemia in mouse models of type 1 diabetes. J Autoimmun 84, 65–74. 28711285 10.1016/j.jaut.2017.07.006

[26] Pfeifle R , Rothe T , Ipseiz N , Scherer HU , Culemann S , Harre U , Ackermann JA , Seefried M , Kleyer A , Uderhardt S *et al* (2017) Regulation of autoantibody activity by the IL‐23‐TH17 axis determines the onset of autoimmune disease. Nat Immunol 18, 104–113. 27820809 10.1038/ni.3579 PMC5164937

[27] Choi JY , Seth A , Kashgarian M , Terrillon S , Fung E , Huang L , Wang LC and Craft J (1950) (2017) Disruption of pathogenic cellular networks by IL‐21 blockade leads to disease amelioration in murine lupus. J Immunol 198, 2578–2588. 10.4049/jimmunol.1601687 PMC5360548 28219887

[28] Mitsdoerffer M , Lee Y , Jager A , Kim HJ , Korn T , Kolls JK , Cantor H , Bettelli E and Kuchroo VK (2010) Proinflammatory T helper type 17 cells are effective B‐cell helpers. Proc Natl Acad Sci USA 107, 14292–14297. 20660725 10.1073/pnas.1009234107 PMC2922571

[29] Coquery CM , Loo WM , Wade NS , Bederman AG , Tung KS , Lewis JE , Hess H and Erickson LD (2015) BAFF regulates follicular helper t cells and affects their accumulation and interferon‐gamma production in autoimmunity. Arthritis Rheumatol 67, 773–784. 25385309 10.1002/art.38950 PMC4342294

[30] Ding Y , Li J , Wu Q , Yang P , Luo B , Xie S , Druey KM , Zajac AJ , Hsu HC and Mountz JD (2013) IL‐17RA is essential for optimal localization of follicular Th cells in the germinal center light zone to promote autoantibody‐producing B cells. J Immunol 191, 1614–1624. 23858031 10.4049/jimmunol.1300479 PMC3819396

[31] Gandhi GR , Neta M , Sathiyabama RG , Quintans JSS , de Oliveira ESAM , Araujo AAS , Narain N , Junior LJQ and Gurgel RQ (2018) Flavonoids as Th1/Th2 cytokines immunomodulators: a systematic review of studies on animal models. Phytomedicine 44, 74–84. 29895495 10.1016/j.phymed.2018.03.057

[32] Liu W , Li WM , Gao C and Sun NL (2005) Effects of atorvastatin on the Th1/Th2 polarization of ongoing experimental autoimmune myocarditis in Lewis rats. J Autoimmun 25, 258–263. 16242301 10.1016/j.jaut.2005.06.005

[33] Vinuesa CG , Linterman MA , Yu D and MacLennan IC (2016) Follicular helper T cells. Annu Rev Immunol 34, 335–368. 26907215 10.1146/annurev-immunol-041015-055605

[34] Gu‐Trantien C , Migliori E , Buisseret L , de Wind A , Brohee S , Garaud S , Noel G , Dang Chi VL , Lodewyckx JN , Naveaux C *et al* (2017) CXCL13‐producing TFH cells link immune suppression and adaptive memory in human breast cancer. JCI insight 2 :e91487 10.1172/jci.insight.91487 PMC5453706 28570278

[35] Kroenke MA , Eto D , Locci M , Cho M , Davidson T , Haddad EK and Crotty S (2012) Bcl6 and Maf cooperate to instruct human follicular helper CD4 T cell differentiation. J Immunol 188, 3734–3744. 22427637 10.4049/jimmunol.1103246 PMC3324673

[36] Weinstein JS , Herman EI , Lainez B , Licona‐Limon P , Esplugues E , Flavell R and Craft J (2016) TFH cells progressively differentiate to regulate the germinal center response. Nat Immunol 17, 1197–1205. 27573866 10.1038/ni.3554 PMC5030190

[37] Xin N , Fu L , Shao Z , Guo M , Zhang X , Zhang Y , Dou C , Zheng S , Shen X , Yao Y *et al* (2014) RNA interference targeting Bcl‐6 ameliorates experimental autoimmune myasthenia gravis in mice. Mol Cell Neurosci 58, 85–94. 24361642 10.1016/j.mcn.2013.12.006

[38] Achour A , Simon Q , Mohr A , Seite JF , Youinou P , Bendaoud B , Ghedira I , Pers JO and Jamin C (2017) Human regulatory B cells control the TFH cell response. J Allergy Clini Immunol 140, 215–222. 10.1016/j.jaci.2016.09.042 27865860

[39] Frohlich A , Marsland BJ , Sonderegger I , Kurrer M , Hodge MR , Harris NL and Kopf M (2007) IL‐21 receptor signaling is integral to the development of Th2 effector responses *in vivo* . Blood 109, 2023–2031. 17077330 10.1182/blood-2006-05-021600

[40] Wang Z , Li M , Zhou M , Zhang Y , Yang J , Cao Y , Wang K , Cui M , Chen H , Fu ZF *et al* (2017) A novel rabies vaccine expressing CXCL13 enhances humoral immunity by recruiting both T follicular helper and germinal Center B Cells. J Virol 91 :e01956–16. 27852854 10.1128/JVI.01956-16 PMC5244327

